# Moving from Challenge to Change: Redesigning Inpatient Care for Children with Complex Care Needs

**DOI:** 10.3390/children13040502

**Published:** 2026-04-02

**Authors:** Emma Popejoy, Jane Coad, Eyal Cohen, Aysha Sheikhi, Joseph C. Manning

**Affiliations:** 1 Nottingham Children’s Hospital, Nottingham University Hospitals NHS Trust, Nottingham NG7 2UH, UK; joseph.manning@leicester.ac.uk; 2 School of Health Sciences, University of Nottingham, Nottingham NG7 2HA, UK; jane.coad@nottingham.ac.uk; 3 Centre for Care Excellence, University Hospitals Coventry and Warwickshire, Coventry CV2 2DX, UK; 4 The Hospital for Sick Children (SickKids), Toronto, ON M5G 1X8, Canada; eyal.cohen@sickkids.ca; 5 Parent Advisor, Birmingham, UK; 6 School of Healthcare, University of Leicester, Leicester LE1 7RH, UK

**Keywords:** children with complex care needs, inpatient care, workforce development, policy drivers

## Abstract

Children with complex care needs represent a growing and highly vulnerable population within inpatient hospital settings. They experience disproportionately long lengths of stay, higher rates of safety incidents, and poorer care experiences than other children. Their increasing prevalence in hospitals reflects broader advances in medical care that have improved survival, yet current inpatient systems remain largely designed around episodic, single-condition models of care. As a result, children with complex care needs and their families frequently encounter inpatient services which are fragmented and stretched and environments which are not adequately suited to their needs. The challenges are well recognised, and current drivers exist to move toward meaningful system change. Recent policy drivers, workforce development agendas, and new funding streams provide an opportunity to reimagine how inpatient services are organised and delivered for this population. This opinion piece, situated within the UK healthcare context, offers a structured analysis of the systemic challenges facing inpatient services for children with complex care needs and identifies priority domains of safe care, workforce development, and knowledge generation, where targeted redesign is both feasible and urgently required.

## 1. Introduction

Children with complex care needs (CCCNs) represent a growing and increasingly visible priority in child health. This population, often described as medically complex and/or fragile, technology-dependent, or living with multiple chronic conditions [1], are identified as having chronic condition(s), functional limitations, high healthcare use and substantial family-identified care needs [2,3]. Many CCCNs require frequent and prolonged interaction with acute care services [4], placing significant strains on and posing challenges for current systems.

Acute care systems have been designed to manage short-term curative care and therefore struggle to meet the needs of children whose conditions demand sustained, multidisciplinary, longitudinal input. While contemporary healthcare systems and policy increasingly emphasise coordinated care in community-based settings, these models have largely evolved in response to the burden of adult multimorbidity [5]. Yet the same principles of person-centred care, quality of life, and integrated support are equally critical for CCCNs [6]. This applies not only in community settings but also within inpatient care. Rising complexity, escalating costs, and workforce pressures illuminate the urgency for innovation and system transformation, particularly in the inpatient setting. Inpatient systems designed around episodic, curative models are increasingly misaligned with the longitudinal, multidisciplinary needs of CCCNs and require deliberate redesign rather than incremental adaptation.

This opinion piece explores systemic challenges in inpatient care for CCCNs and highlights opportunities for change as a result of global policy, workforce development and funding priorities. Whilst some of the challenges and opportunities outlined within this paper may be relevant to community or rehabilitation settings, this piece focuses solely on inpatient care, defined as overnight stays in acute hospital settings. Though grounded in the United Kingdom (UK) context, these issues may resonate across health systems in high-resource contexts, where similar demographic and structural pressures persist. Given substantial differences in funding models, workforce structures, digital infrastructure, and case mix across high-resource systems, we do not specify the conditions required for transferability. Instead, readers are encouraged to consider the relevance of these issues in relation to their own system’s resources, staffing profile, and the levels of clinical complexity they manage.

## 2. Challenges with Inpatient Care

To contextualise the inpatient experiences of CCCNs, a vignette is presented. This highlights how, through a cumulative effect, these hold greater significance for CCCNs than for children without complex care needs. The specific challenges associated with inpatient care will then be described in more depth.

### 2.1. Vignette

Molly is a two-year-old girl with complex care needs who has been an inpatient for five months following recurrent chest infections and seizures. During this admission, investigations identified sleep-disordered breathing and discussions regarding longer-term respiratory support, either non-invasive ventilation (NIV) or tracheostomy, were initiated.

Reaching a decision about the most appropriate management pathway proved challenging. Molly’s care was shared between Neurology, Respiratory, Gastroenterology and Ear, Nose and Throat teams, none of whom routinely reviewed her together. Documentation was inconsistent, and communication across teams was sub-optimal, contributing to delays in achieving a unified plan. After five months of admission and an unsuccessful trial of NIV, a multidisciplinary team meeting was eventually convened, where consensus was reached to proceed with tracheostomy. A theatre date was arranged for the following week.

Molly was cared for in an open bay on a ward that also accommodated several other CCCNs. The workload was high, with numerous patients requiring complex medication regimes, time-critical interventions and substantial liaison between teams and with external professionals, such as community nurses and social care teams. This high overall workload meant that routine and time-dependent care activities were frequently delayed. On several occasions, this included delays to Molly’s own time-critical anticonvulsant medications, further contributing to instability in her seizure control.

In the days leading up to surgery, Molly acquired RSV from another patient in the bay, leading to the cancellation of her theatre slot. This change was not adequately handed over, and she was fasted unnecessarily, remaining without nutrition for six hours. This was significant, given ongoing challenges in maintaining her weight. As her infection worsened, she experienced an escalation in seizures, including a prolonged episode requiring Paediatric Intensive Care Unit admission.

Molly’s parents were distressed by her deterioration and the repeated communication breakdowns. They had previously requested that Molly be moved to a side room, recognising her vulnerability to infection and the importance of maintaining her surgical pathway, but this had not been actioned. Their concerns grew as fragmented communication, inconsistent care delivery and competing ward pressures continued to impact upon Molly’s hospital journey.

This vignette illustrates how routine system pressures, such as fragmented communication, unclear leadership, and high ward workloads, can combine to create significant clinical and emotional consequences for CCCNs and their families. These challenges are now described in depth.

### 2.2. Child- and Family-Centred Care

Traditionally, hospital systems were designed to manage short-term acute illnesses or injury in otherwise healthy children, discharging them once they recovered [7]. In this model, families had little involvement in care and healthcare professionals were regarded as the primary experts, making decisions and delivering treatment with minimal parental/carer input. However, the growing population of CCCNs does not fit this mould. Their parents/carers frequently possess advanced knowledge and skills, performing invasive procedures daily and holding unparalleled expertise in their child’s condition, needs and clinical baseline. All parents provide essential insights into their child’s baseline behaviour and needs during hospitalisation. However, parents of CCCNs often possess additional clinical-level skills, including detailed knowledge of medication dosing and interactions, experience with invasive devices, and expertise in recognising early signs of deterioration. These competencies mean their involvement is not only valuable but critical to safe, effective inpatient care.

This epidemiologic shift challenges traditional ways of working. While policy promotes shared decision-making and family-centred care, this often does not translate into practice for CCCNs [8], where structural constraints, time pressures, and entrenched practices often prevent these ideals from being realised. As a result, tensions can arise, eroding trust between families and professionals. Fragmented, system-focused care compounds these issues, with children frequently treated as a collection of organ systems rather than as whole individuals. For CCCNs, delays, communication failures, or fragmented decision-making often accumulate across multiple specialties and episodes of care, leading to more frequent safety incidents, longer admissions, and greater disruption to family life than typically experienced by children without complex care needs. Parents/carers often become the primary coordinators, relaying critical information between siloed teams [9], a role associated with immense emotional and cognitive strain. The errors and delays in care [10,11,12,13,14] serve to reinforce parent/carers’ perception that they must remain constantly at the bedside to safeguard their child. This sustained vigilance is associated with physical exhaustion, mental distress, and significant disruption to family life. Consequently, families of CCCNs consistently report poorer experiences of hospital care [15].

### 2.3. Increasing Needs, Limited Capacity

Reflection on inpatient caseloads over the past two decades, across multiple organisations and countries, reveals a striking shift in the paediatric population. Twenty years ago, medical complexity was rare and often less severe. Many CCCNs back then did not survive because the knowledge and technology to sustain them simply did not exist. Advances in medicine and clinical expertise mean children who once would not have lived now survive, often with extreme complexity [16] and profound, multifaceted needs.

This evolution has placed immense pressure on a system that has not adequately adapted to its changing population. Increasing medicalisation and technological dependence mean that children often require highly specialised interventions and equipment, adding layers of complexity to care delivery. At the same time, hospitals face relentless throughput demands and persistently sub-optimal staffing levels [7,17,18], leaving health professionals with limited time to build relationships, understand family dynamics, and provide holistic, compassionate care. Additionally, professionals have identified that lack of time allocated to clinicians to deliver holistic care for CCCNs and lack of appropriate pathways through the system are barriers to optimal, family-centred care for CCCNs [19]. These pressures create a clinical reality that stifles innovation. Professionals have limited capacity to reimagine how services could be delivered differently, and there remains a relative paucity of research focused on improving inpatient care for CCCNs [20,21]. Multidisciplinary teams are frequently unable to deliver the comprehensive and specialised care that CCCNs and their families need. These challenges frequently lead to prolonged hospital stays and delayed discharges, amplifying system strain while placing significant physical, emotional, and financial burdens on families.

### 2.4. Cost

Hospital care is inherently costly. Emerging evidence from multiple countries also demonstrates a marked concentration of CCCN care within inpatient settings. In the UK, the inpatient costs for the high-cost high-need group, defined as the top 5% of paediatric healthcare spending (including, but not limited to, CCCNs), account for 34% of total healthcare spending [22]. In a Canadian study, CCCNs accounted for <1% of the overall paediatric population but 23% of all hospital admissions and 52% of total inpatient costs ($2.25 billion) [23]. Another American study found that inpatient costs for CCCNs were 17 times greater than those for children without chronic health needs [24]. Collectively, these data illustrate the disproportionate concentration of paediatric healthcare expenditure within inpatient services for children with complex needs. Interventions to deliver coordinated care in the community are the subject of increasing research and are vital to reducing these costs and providing optimal quality of life for CCCNs and their families. However, the underlying nature of these children’s conditions means that, despite optimal community provision, frequent hospital admissions are often unavoidable.

## 3. A Call for Action

The challenges and associated vignette outlined demonstrate that it is essential to work towards change. Clinicians, hospital administrators, and policy-makers must partner with patients and families to improve how inpatient care is delivered, creating better experiences, ensuring coordinated care, and strengthening communication to deliver cost-effective care, minimising errors and achieving timely discharge for hospitalised CCCNs. The evidence outlined makes it clear why this is urgently needed. The trend toward increasing complexity in paediatric populations shows no sign of slowing [25], so we must adapt our systems now to meet the challenges ahead.

## 4. Opportunities for Improvement

Using the UK as an example, there are some key policy, workforce and safety drivers which present opportunities for improvements in the care of CCCNs. These are underpinned by the National Health Service (NHS) 10 Year Health Plan [26], which highlights the importance of personalisation, reducing health inequalities and improving outcomes for children and young people. Opportunities for improvement largely fall under three categories: (1) delivering safe, quality care; (2) strengthening the workforce; (3) advancing innovation and knowledge generation.

### 4.1. Opportunities for Delivering Safe, Quality Care for CCCNs

Families of CCCNs are not passive recipients of care: they are often expert partners whose knowledge and advocacy can significantly enhance safety and quality. Opportunities at this level include co-designing care plans/services, implementing family-held records or digital care passports, and establishing structured family involvement in ward rounds and discharge planning. Training families in medication administration, infection prevention, and escalation protocols can reduce harm and improve confidence. Peer support networks and family advisory councils offer platforms for shared learning and influence on service design. Recognising and supporting the emotional and practical burden on families through respite care, psychological support, and financial advice is equally critical. Empowering families as partners in safety and decision-making is fundamental to delivering high-quality care for CCCNs. Although some such strategies are well described in broader paediatric practice, their application within inpatient care for CCCNs has not been fully established. Recent research has explored approaches to enhancing quality and safety for inpatient CCCNs through patient- and family-centred care [27], parent/carer safety reporting [28] and medication safety rounding [29]. However, these and other novel, complex interventions remain in need of investigation to demonstrate their effectiveness for CCCNs. While promising, at present they do not represent established solutions.

Engaging with children, parents/carers and families was highlighted globally as one of the World Health Organisation’s goals for paediatric patient safety in 2025. This internationally recognised driver for change has specific relevance to CCCNs [30], as the other goals focus on: medication safety; diagnostic safety; healthcare-associated infections; and reducing risk for small/sick newborns. While attention to these safety goals provides a valuable strategic framework for improving inpatient care for CCCNs and, more broadly, for all children accessing acute services, the publication of these goals alone will not drive meaningful change. When coupled with targeted research investment and potential changes in workforce, however, they offer a timely and robust foundation for initiating sustainable improvements in paediatric inpatient care for CCCNs.

### 4.2. Opportunities for Workforce Improvements

CCCNs require a multidisciplinary and multi-speciality approach to care. Inpatient teams are beginning to recognise the value of parent and carer expertise, but education is needed to equip professionals with the skills to harness this effectively. Whilst such training exists in some settings, it is not yet mandatory. Embedding it as a core element of paediatric healthcare education, rather than an optional extra, would help create a culture where meaningful engagement with families becomes the norm.

Encouragingly, current policy shifts, including the UK NHS 10 Year Health Plan [26], provide a strong platform for this change. Its emphasis on person-centred teams aligns with preventing fragmentation of care. Alongside this, there is growing focus on the use of interprofessional simulation training [31] to enhance quality and safety of care. These policy and guidance documents set the context in which there exists a fantastic opportunity to establish interprofessional simulation training to support healthcare teams to develop the necessary skills for communicating with CCCNs and their expert families. Advanced practice roles are also advocated in the NHS 10 Year Health Plan [26] and could enable allied health professionals to take on key responsibilities for care coordination.

In the inpatient setting, nurses, midwives and allied health professionals are uniquely positioned to lead improvements in care for CCCNs. Their proximity to the child and family provides a critical lens on both the practical realities of care delivery and the relational factors that underpin the family’s experience. Expanding research capacity and capability within these professional groups offers a powerful opportunity to reimagine inpatient care for CCCNs, as when they are given the space and support to engage in research, their insight becomes a powerful tool for overcoming barriers to improvement. Dedicated funding streams for underrepresented healthcare disciplines [32] provide an opportunity for professionals to enhance their academic skills, knowledge and experience through protected time, mentorship and career development, while maintaining hands-on patient care. In addition, increasing attention to non-medical clinical academic roles in the UK creates the conditions for the advancement of this career pathway. Embedding such roles for CCCNs within hospital settings would help bridge practice and research, amplify the patient voice in research proposals and empower frontline staff to innovate and influence policy.

### 4.3. Opportunities for Evidence Generation and Knowledge Exchange

Robust, multidisciplinary research is critical to addressing the complexity of care for CCCNs. Breaking down silos and fostering collaboration ensures that research reflects diverse perspectives and operational realities. Strategic funding priorities, such as frameworks for multiple long-term conditions [6], create opportunities to develop and evaluate innovative models of acute care. Implementation science methods and learning health systems can accelerate translation of evidence into routine inpatient practice. Knowledge exchange should be active and inclusive, using communities of practice, open-access toolkits, and digital platforms to share safety dashboards and family-facing resources. Embedding nurses, midwives and allied health professionals in research leadership will amplify patient-centred insights and ensure that evidence informs policy and practice.

### 4.4. Drive Towards Digital Innovation

Another key aspect of the UK NHS 10 Year Health Plan [26] is its emphasis on digital transformation, which presents a further opportunity to improve inpatient care for CCCNs. The digital revolution offers the potential for innovations to enhance continuity of information for CCCNs amongst ever-changing inpatient healthcare teams. Digital tools, such as secure messaging, telehealth consultations, and symptom tracking apps, can strengthen communication and continuity between hospital and home. They can also be harnessed within the acute inpatient environment to enhance care coordination, peer support and communication between the family and wider teams, thereby offering real potential for improved experiences and outcomes during hospital admissions.

Despite the existing challenges that exist in the system, there are a number of key facilitators (notably funding opportunities) indicating that now is the time for change. By working collaboratively across disciplines, we can capitalise on these opportunities and ensure that when CCCNs, like Molly, are admitted to hospital, they receive the best possible outcomes and overall experience.

## 5. A Rising Tide Lifts All Boats

Importantly, the benefits of improving care for CCCNs extend well beyond this group. Despite the increase in bed occupancy among CCCNs, there will always be children without complex care needs who are admitted to hospital with acute illness or injury and require timely, curative treatment. Designing systems that deliver excellent care for CCCNs will not detract from the care received by children without complex care needs; rather, the enhancements needed for CCCNs, such as stronger family-centred care, clearer communication, and an enhanced focus on safety, will elevate the quality of care for every child and family accessing hospital services. Addressing these systemic issues for CCCNs will create more reliable, coordinated, and compassionate inpatient environments that benefit every child and family accessing hospital services.

## 6. Conclusions

Although significant barriers persist in delivering optimal inpatient care for CCCNs, recent UK policy developments, workforce investment, and targeted funding create a timely opportunity to redesign inpatient systems to better align with the needs of this population. To realise this potential, we must draw on global safety frameworks, leverage national priorities, and capitalise on emerging investment in clinical–academic career development across disciplines. Multidisciplinary collaboration, inclusive research practices, and the deliberate dismantling of speciality and organisational silos will be essential to achieving tangible improvements in care quality and the inpatient experience for CCCNs. The opportunity is here and the momentum is growing. The time is ripe for collective action.

## Abbreviations

The following abbreviations are used in this manuscript:

CCCNs	Children with complex care needs
UK	United Kingdom
NHS	National Health Service

## References

[1] Brenner M., Greene J., Doyle C., Koletzko B., Del Torso S., Bambir I., De Guchtenaere A., Polychronakis T., Reali L., Hadjipanayis A.A. (2021). Increasing the Focus on Children’s Complex and Integrated Care Needs: A Position Paper of the European Academy of Pediatrics. Front. Pediatr..

[2] Cohen E., Kuo D.Z., Agrawal R., Berry J.G., Bhagat S.K.M., Simon T.D., Srivastava R. (2011). Children with Medical Complexity: An Emerging Population for Clinical and Research Initiatives. Pediatrics.

[3] Millar K., Rodd C., Rempel G., Cohen E., Sibley K.M., Garland A. (2024). The Clinical Definition of Children with Medical Complexity: A Modified Delphi Study. Pediatrics.

[4] Leyenaar J.K., Freyleue S., Arakelyan M., Schaefer A.P. (2025). Hospitalizations by Children with Medical Complexity from 2009 to 2022. Pediatrics.

[5] The Academy of Medical Sciences (2018). Multimorbidity: A Priority for Global Health Research.

[6] National Institute for Health and Care Research (2024). NIHR Strategic Framework for Multiple Long-Term Conditions (Multimorbidity) MLTC-M Research.

[7] Cass H., Barclay S., Gerada C., Lumsden D.E., Sritharan K. (2020). Complexity and challenge in paediatrics: A roadmap for supporting clinical staff and families. Arch. Dis. Child..

[8] Yun H.J., Parker M.L., Wilson C.B., Cui M. (2024). Medical Complexity of Children with Special Healthcare Needs and Healthcare Experiences. Children.

[9] Cady R.G., Belew J.L. (2017). Parent Perspective on Care Coordination Services for Their Child with Medical Complexity. Children.

[10] DeCourcey D.D., Silverman M., Chang E., Ozonoff A., Stickney C., Pichoff D., Oldershaw A., Finkelstein J.A. (2017). Medication Reconciliation Failures in Children and Young Adults with Chronic Disease During Intensive and Intermediate Care. Pediatr. Crit. Care Med..

[11] Ahuja N., Zhao W., Xiang H. (2012). Medical errors in US pediatric inpatients with chronic conditions. Pediatrics.

[12] Nackers A., Ehlenbach M., Kelly M.M., Werner N., Warner G., Coller R.J. (2019). Encounters from Device Complications Among Children with Medical Complexity. Hosp. Pediatr..

[13] Mercer A.N., Mauskar S., Baird J., Berry J., Chieco D., Copp K., Cox E.D., Haskell H., Hennessy K., Kelly M.M. (2022). Family Safety Reporting in Hospitalized Children with Medical Complexity. Pediatrics.

[14] Fox S.C., Marufu T.C., Popejoy E., Taylor N., Roland D., Manning J.C. (2026). Causes, characteristics and improvement strategies for delayed interventions during paediatric deterioration: A mixed methods systematic review. Intensive Crit. Care Nurs..

[15] Care Quality Commission National Children’s Hospital Survey Finds Most Children Have Good Experiences of Care—But Highlights Inequalities for Those with Specific Needs. https://www.cqc.org.uk/news/releases/national-childrens-hospital-survey-finds-most-children-have-good-experiences-care-%E2%80%93.

[16] Rogers J., Reed M.P., Blaine K., Manning H. (2021). Children with medical complexity: A concept analysis. Nurs. Forum.

[17] Barrett Fromme H., Jackson K., Cook J.A., Birnie K.L., Alcon S., Kuhn E.C., Dawlett M., Mike T.B. (2025). Insights into the Pediatric Hospital Medicine Workforce: 2024 Data and Recent Trends for Programs. Hosp. Pediatr..

[18] OECD/European Commission (2024). Health at a Glance: Europe 2024: State of Health in the EU Cycle.

[19] McLorie E.V., Fraser L., Hackett J. (2023). Provision of care for children with medical complexity in tertiary hospitals in England: Qualitative interviews with health professionals. BMJ Paediatr. Open.

[20] Popejoy E., Coad J., Cohen E., Pearson A., Williams R., Manning J.C. (2025). Enhancing the experience and outcomes of children with complex care needs in acute paediatric settings: A realist review protocol. BMJ Open.

[21] Dewan T., Mackay L., Asaad L., Buchanan F., Hayden K.A., Montgomery L. (2024). Experiences of Inpatient Healthcare Services Among Children with Medical Complexity and Their Families: A Scoping Review. Health Expect..

[22] Punjabi N., Marszalek K., Beaney T., Shah R., Nicholls D., Deeny S., Hargreaves D. (2022). Categorising high-cost high-need children and young people. Arch. Dis. Child..

[23] Hessey E., Thavam T., Mahant S., Cohen E., Zhu J., Buchanan F., To T., Gill P.J. (2025). Most costly and prevalent reasons for hospitalization in children with medical complexity in Ontario, Canada. J. Hosp. Med..

[24] Kuo D.Z., Melguizo-Castro M., Goudie A., Nick T.G., Robbins J.M., Casey P.H. (2015). Variation in child health care utilization by medical complexity. Matern. Child. Health J..

[25] Feudtner C. (2025). Medical Complexity Is an Unintended Consequence of Medical and Surgical Success. JAMA Netw. Open.

[26] Department of Health and Social Care (2025). Fit for the Future: 10 Year Health Plan for England.

[27] Rennick J.E., Southall K., Carnevale F., Dryden-Palmer K., Patel H., Dagenais M., Buchanan F., Avery S., Razack S., St-Sauveur I. (2025). Using experience-based co-design to explore care experiences and identify practice change priorities for children with medical complexity in the paediatric intensive care unit. BMJ Open.

[28] Khan A., Baird J., Mauskar S., Haskell H.W., Habibi A.N., Ngo T., Aldarondo A., Berry J.G., Copp K.L., Liu J.P. (2024). A Coproduced Family Reporting Intervention to Improve Safety Surveillance and Reduce Disparities. Pediatrics.

[29] Rojas C.R., Moore A., Coffin A., McClam C., Ehritz C., Hogan A., Hart J., Galligan M.M. (2023). Medication Rounds: A Tool to Promote Medication Safety for Children with Medical Complexity. Jt. Comm. J. Qual. Patient Saf..

[30] World Health Organization (2025). World Patient Safety Day Goals 2025.

[31] Health Education England (2018). National Framework for Simulation Based Education.

[32] National Institute for Health and Care Research (2021). Best Research for Best Health: The Next Chapter.

